# Publication of Medical Books

**Published:** 1863-05

**Authors:** 


### PUBLICATION OF MEDICAL BOOKS.

The activity and enterprise of our medical book publishers is quite astonishing, who, notwithstanding the vastly augmented expense of publication are yet sending out to the medical public the most elaborate and expensive works. For a few months after the outbreak of the rebellion and the advance in all the materials of book manufacture, we were not favored with the larger and more valuable works or with new editions of the same. It appeared that the physicians had many of them "gone to the war," and those remaining were not in situation to patronize book merchants. The commercial interests of the country temporarily suffered great oppression, and this interest of all others, seemed likely to suffer most. We call attention to this subject since it indicates so much for the medical profession; shows the prosperity of physicians and general advance of medical science, telling plainly and unmistakably the true condition of the medical pocket as well as medical mind; and indicating the general prosperity of the North while yet bearing the burdens of war. It will be observed that we have received within the last few days valuable new books, and new editions of books, which have been standard works, and are widely known and highly appreciated by the profession. These books are from the publishing houses of *Lindsay & Blakiston*, *Blanchard & Lea*, *J. B. Lippencott & Co.*, *Harper & Brothers*, and *William Wood*, and will be noticed at length as soon as time will permit; since all our readers will be interested to know something of their character.

